# Molecular Cloning, Characterization and Predicted Structure of a Putative Copper-Zinc SOD from the Camel, *Camelus dromedarius*

**DOI:** 10.3390/ijms13010879

**Published:** 2012-01-16

**Authors:** Farid S. Ataya, Dalia Fouad, Ebtsam Al-Olayan, Ajamaluddin Malik

**Affiliations:** 1 Department of Biochemistry, College of Science, King Saud University, P.O. Box 2455, Riyadh 11451, Saudi Arabia; 2 Department of Molecular Biology, Genetic Engineering Division, National Research Center, Dokki, Cairo, 12311, Egypt; 3 Department of Zoology, College of Science, King Saud University, P.O. Box 22452, Riyadh 11459, Saudi Arabia; E-Mails: dibrahim@ksu.edu.sa (D.F.); eolayan@ksu.edu.sa (E.A.-O.); 4 Protein Research Chair, Department of Biochemistry, College of Science, King Saud University, P.O. Box 2455, Riyadh 11451, Saudi Arabia; E-Mail: amalik@ksu.edu.sa

**Keywords:** CuZnSOD, 3D structure, cloning, molecular characterization, one-humped camel

## Abstract

Superoxide dismutase (SOD) is the first line of defense against oxidative stress induced by endogenous and/or exogenous factors and thus helps in maintaining the cellular integrity. Its activity is related to many diseases; so, it is of importance to study the structure and expression of *SOD* gene in an animal naturally exposed most of its life to the direct sunlight as a cause of oxidative stress. Arabian camel (one humped camel, *Camelus dromedarius*) is adapted to the widely varying desert climatic conditions that extremely changes during daily life in the Arabian Gulf. Studying the cSOD1 in *C. dromedarius* could help understand the impact of exposure to direct sunlight and desert life on the health status of such mammal. The full coding region of a putative CuZnSOD gene of *C. dromedarius* (*cSOD1*) was amplified by reverse transcription PCR and cloned for the first time (gene bank accession number for nucleotides and amino acids are JF758876 and AEF32527, respectively). The cDNA sequencing revealed an open reading frame of 459 nucleotides encoding a protein of 153 amino acids which is equal to the coding region of *SOD1* gene and protein from many organisms. The calculated molecular weight and isoelectric point of cSOD1 was 15.7 kDa and 6.2, respectively. The level of expression of *cSOD1* in different camel tissues (liver, kidney, spleen, lung and testis) was examined using Real Time-PCR. The highest level of *cSOD1* transcript was found in the camel liver (represented as 100%) followed by testis (45%), kidney (13%), lung (11%) and spleen (10%), using 18S ribosomal subunit as endogenous control. The deduced amino acid sequence exhibited high similarity with *Cebus apella* (90%), *Sus scrofa* (88%), *Cavia porcellus* (88%), *Mus musculus* (88%), *Macaca mulatta* (87%), *Pan troglodytes* (87%), *Homo sapiens* (87%), *Canis familiaris* (86%), *Bos taurus* (86%), *Pongo abelii* (85%) and *Equus caballus* (82%). Phylogenetic analysis revealed that cSOD1 is grouped together with *S. scrofa*. The predicted 3D structure of cSOD1 showed high similarity with the human and bovine CuZnSOD homologues. The Root-mean-square deviation (rmsd) between cSOD1/hSOD1 and cSOD1/bSOD1 superimposed structure pairs were 0.557 and 0.425 A. The *Q-*score of cSOD1-hSOD1 and cSOD1-bSOD1 were 0.948 and 0.961, respectively.

## 1. Introduction

Domesticated Arabian camel, *Camelus dromedarius*, is the most important animal in the Arabian Desert, as it represents the main source of meat and its milk and urine are used for the maintenance of health and the treatment of various diseases [1], besides its high cultural and economical values. This animal, like other living organisms, is continuously exposed to deleterious endogenous and exogenous factors that results in a disturbance in the balance between the production of ROS and antioxidant defenses, causing DNA damage [2,3]. Mammalian cells are equipped with both enzymatic and non-enzymatic antioxidant defense mechanisms to minimize the cellular damage resulting from the interaction between cellular constituents and ROS. The enzymatic antioxidant mechanism contains various forms of superoxide dismutase (SOD), catalase and glutathione peroxidase [4].

SOD is widespread in nature especially in all oxygen-metabolizing cells. It represents the first line of defense against the potentially damaging reactivities of the superoxide radical O_2_^−^, generated by aerobic metabolic reactions [5]. It scavenges O_2_^−^ anion and converts them into H_2_O_2_ and O_2_. Although H_2_O_2_ is not itself a free radical it can be toxic at high concentrations, so it is broken down into water by catalase in peroxisomes and glutathione peroxidase in the cytosol and mitochondria [4]. SOD has been purified from diverse sources such as: fungi [6]; bacteria [7]; yeast [8]; plants [9] and animals [10,11].

Three SOD enzymes are characterized by different metal content in their active site: blue-green copper and zinc (CuZnSOD or SOD1), wine-red manganese (Mn-SOD) and yellow iron (Fe-SOD). SOD1 is found widely in the cytoplasm and in the mitochondrial intermembrane space of the eukaryotic cells [12]. Mn-SOD are located in prokaryotes and in the mitochondrial matrix of eukaryotes [13], while Fe-SOD has been found in bacteria, blue-green algae and protozoa [7,14]. Generally, mammals have three distinct types of SODs; the SOD1 is predominantly expressed in the cytosol, the MnSOD is located in the mitochondria of the cells [13] and extracellular SOD (EC-SOD) is present in the intravascular and extracellular fluids such as plasma, lymph, and synovial fluid [12]. SOD1 consists of two subunits of identical molecular weight. It has two Cu(II) and two Zn(II) atoms per molecule. Zinc has a structural, stabilizing role, while Cu^2+^ is directly involved in the catalytic activity [15]. The removal of both zinc and copper yields the apoenzyme [16,17].

The O^2−^ ion has been considered important in aging, lipid peroxidation and the peroxidative hemolysis of red blood cells [18]. SOD1 genetic polymorphisms and altered gene expressions and/or enzyme activities are associated with oxidative DNA damage and cancer susceptibility [19]. Although some point mutations in the SOD1 cause the fatal neurodegenerative disease familial myotrophic lateral sclerosis [20–22], its overexpression is linked to the neural disorders in Down syndrome [23] and is associated with the tolerance of spermatogonia toward the ROS [24]. It has been reported that *SOD1*-deficient mice showed drusen formation, which is a typical characteristic of age-related macular degeneration [25], fatty liver [26], skin thinning [27], symptoms of hepatic carcinoma [28], hemolytic anemia [29], muscle atrophy [30] and reduced fertility in mice [31,32].

The Arabian camel spends most of its life in drought, heat, direct exposure to sunlight, and to many other endogenous and exogenous xenobiotics which result in the production of ROS. It is well adapted to such harsh desert conditions. So, it proposed that camel could have robust mechanisms for eliminating ROS. To date, no researches have been done to identify and clone camel *SOD1* gene. The aim of the present work was to isolate *C. dromedarius* full-coding region corresponding to *SOD1* and to study the degree of similarity of the deduced protein with those of other mammals. In a recent study, we sequenced, cloned and analyzed the first *C. dromedarius SOD1* gene, studied its expression on the level of the transcript by qPCR in five tissues, analyzed the structure, stability and function of *C. dromedarius* SOD1 by multiple sequence analysis and structural superimposition of 3D structure homologous human and bovine SOD1. This strategy has been used to study protein families and in elucidation of the role of conserved amino acid residues in the structure, stability and biological activity of the proteins.

## 2. Results

### 2.1. Cloning and Characterization of Full Coding of *cSOD1* Gene

**Figure 1:**
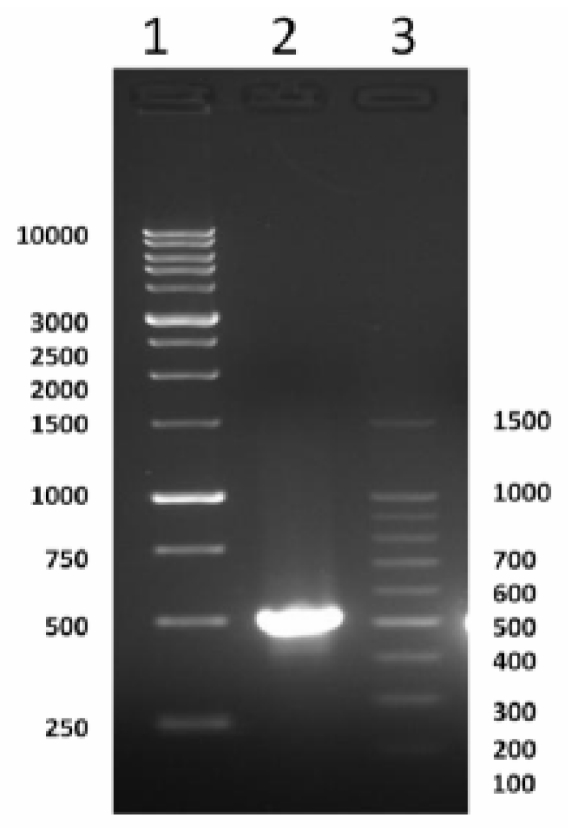
Agarose gel (1.5%) electrophoresis of PCR product of *Camelus dromedarius SOD1* (Lane 2). Lane 1 and 3 contain 1 kb and 100 bp DNA molecular weight marker, respectively.

**Figure 2:**
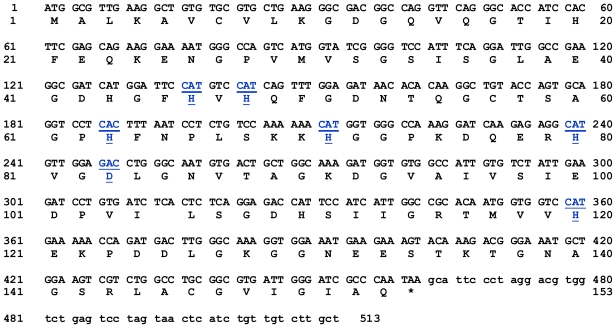
The nucleotide sequence and the deduced amino acids of the cloned *cSOD1*. The sequences were submitted to NCBI GenBank (accession number JF758876 and AEF32527, respectively). The metal binding residues and its corresponding codons are underlined.

A PCR-based technique was used in order to isolate the full length of *cSOD1*. Specific primers were designed from the most conserved region of the available sequencing data in GenBank. A cDNA fragment of 513 bp was amplified by RT-PCR. The optimum annealing temperature was 58 °C. The amplified cDNA was separated by electrophoresis on 1.5% agarose gel which showed the expected band size comparing with the standard molecular weight ladder (Figure 1). This fragment was cut from the agarose gel and purified by gel clean, ligated in pGEM-T Easy plasmid vector and cloned in *E. coli.* The positive clones were selected by blue and white colony using LB/IPTG/X-gal/Ampicillin/agar plates. The white colonies were picked and subjected to colony PCR to ensure the presence of the insert and the plasmid was purified from liquid medium. The insert was sequenced using T7 and SP6 primers. The sequence indicated that the fragment has a length of 513 bp (Figure 2). This sequence represented the first cloned SOD1 from camel. It covers the full coding region comparing with the corresponding regions from different organisms. Our sequence was submitted in the gene bank (accession number JF758876). The deduced amino acid sequence of cSOD1 was found to consist of an open reading frame of 153 amino acid residues (Figure 2). The amino acid sequence was submitted in the gene bank (accession number AEF32527). The BLAST analysis for the coding region of *cSOD1* showed that it shared high similarity (90–89%) with *SOD1* from other mammals (90% marmoset, 90% tufted capuchin, 90% pig, 90% white-cheeked gibbon, 89% Rhesus monkey, 89% cynomolgus monkey, 89% human, 89% guinea pig, 89% chimpanzee, 88% dog, 88% panda, and 88% cattle).

### 2.2. Amino Acid Composition and Protein Secondary Structure

The molecular analysis of the 153-amino acid sequence of cSOD1 using the program PROTEAN [33] showed that this protein has a molecular weight of 15.74 KDa and pI 6.23. The predicted protein contains 34 charged amino acids (28.1%), 44 hydrophobic (28.76%), 18 acidic (11.76%), 13 basic (8.5%) and 34 polar amino acids (22.22%). The complete amino acid analysis and chemical composition of the predicted protein are illustrated in Table 1.

**Figure 3:**
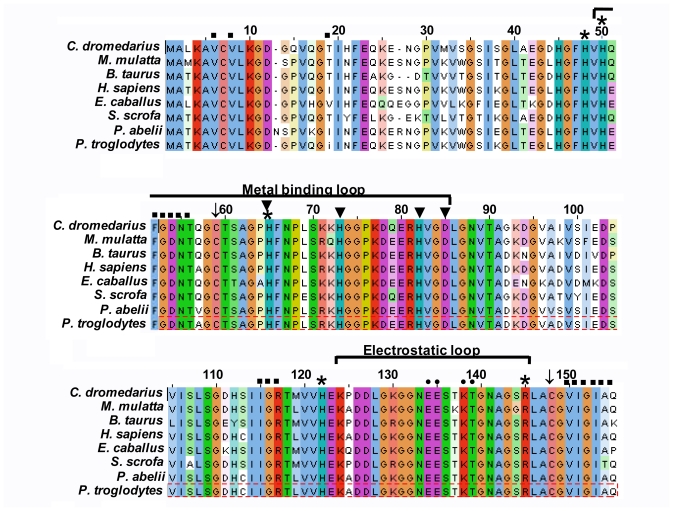
Amino acid sequence alignment of cSOD1 with seven different mammalian proteins. The alignment was generated with the MAFFT Multiple Sequence Alignment program [34]. Residues are color coded according to their conservancy. The Cu and Zn binding site is present on 36 amino acids long stretch of flexible loop. The highly conserved copper binding residues are labeled by (*) and zinc binding residues are labeled by (▾). Conserved His 63 facilitates the binding of both Cu and Zn. Mammalian SOD1 exits as dimer which is formed by hydrophobic (shown by ■) and electrostatic (shown by ●) interactions.

**Figure 4:**
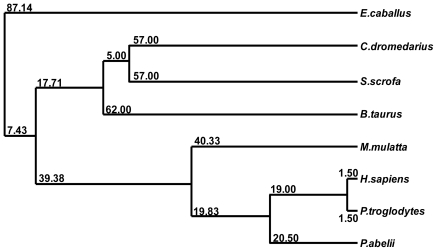
The phylogenetic tree of cSOD1 and potentially related genes. The protein sequence of Camel SOD1 was compared with other mammalian sequences of the GenBankTM data base. The alignment was generated with the BLOSUM62 from MAFFT Multiple Sequence Alignment [34,36]

**Figure 5:**
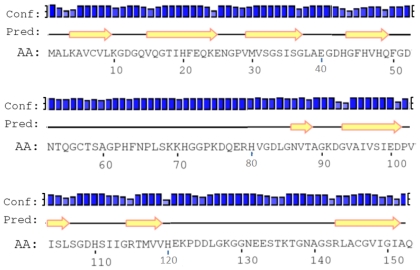
The secondary structure annotation sites of the cSOD1 sequence using PSIPRED program [35]. Yellow arrows indicated β-sheets.

The comparison between the predicted amino acid sequence of cSOD1 and the sequences from the best characterized representatives of SOD1 from different organisms was carried out. The BlastP analysis showed that cSOD1 shared high similarity with SOD1 from different mammalian species. The highest similarity was found with pig *S. scrofa* (88%), Rhesus monkey *M. mulatta* (87%), chimpanzee *P. troglodytes* (87%), human *H. sapiens* (87%), cattle *B. taurus* (86%), Sumatran orangutan *P. abelii* (85%) and horse *E. caballus* (82%), respectively (Table 2, Figure 3). Such high similarity proposed a close evolutionary relationship. The phylogenetic tree of the examined proteins indicated that this cSOD1 groups with *S. scrofa* (Figure 4). A prediction of the secondary structure analysis of cSOD1 was carried out using PSIPRED program [35] (Figure 5). The predicted structure suggested that this protein is composed of 9 β-sheets.

### 2.3. Multiple Sequence Alignment

The amino acid sequence of cSOD1 was aligned with seven different mammalian SOD1 by ClustalW [34,36] (Figure 3). The disulfide cysteins (Cys 57 and Cys 146) responsible for the stability of SOD’s are highly conserved in all compared proteins. The 36 amino acids long metal binding loop is disulfide bonded (Cys 57–Cys 146) with β8 sheet of β-barrel which intern stabilizes the flexible catalytic loop. Comparing with different SOD1, cSOD1 is supposed to be formed from a dimer of two identical subunits. Each monomer could have two metal ions (Cu and Zn). The binding of Cu and Zn plays structural and catalytic roles in SOD. The catalytic Cu is liganded with four surface exposed conserved histidines (H46, 48, 63 and 120) and the Zn is liganded with three buried conserved histidines (H63, 71 and 80) and one conserved Asp83 (Figure 3).

The mammalian SOD’s are stable dimer which is held together by mainly conserved hydrophobic residues (Figure 3). Highly conserved electrostatic residues located on the electrostatic loops makes upper rim of catalytic site (Figure 3). These electrostatic residues shape as well as maintain the electrostatic potential around active site [37].

### 2.4. 3D Structure Prediction

**Figure 6:**
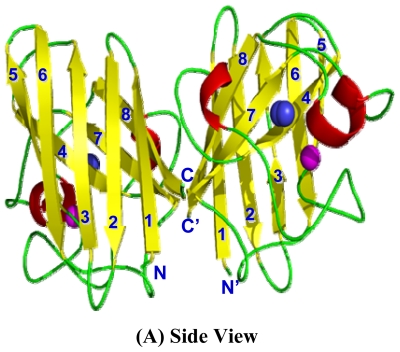

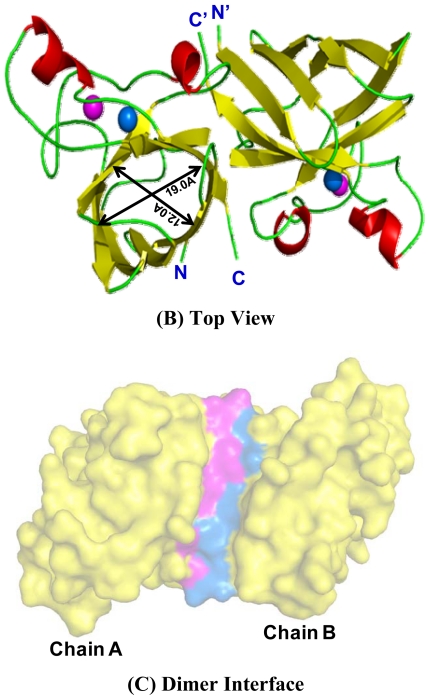
Predicted 3D structure model of cSOD1 and the dimer interface structure. The 3D structure model of cSOD1 was predicted using Swiss-model server [40]. (**A**) Each dimer contains 8 beta sheets. Active site metal ions are shown as spheres for copper (blue) and zinc (purple); (**B**) Top view of cSOD1 reveals the β-barrel structure with 19.0 × 12.0 Å pore; (**C**) Dimer contact forming residues in chain A and B of cSOD1 are shown in magenta and marine colors, respectively.

The 3D structure of cSOD1 was predicted using homology structure modeling on Swiss model server [38]. The 3D structure of human-mouse SOD1 chimera (PDB ID 3gtvE) at 2.2 Å resolution with 88.24% sequence identity was used as a template to predict the 3D structure of cSOD. The predicted 3D structure of cSOD1 reveals overall folding and secondary structures very similar to those of *H. sapiens* (Figure 6). The 3D structure of camel SOD1 is an eight stranded Greek-key β-barrel dimeric protein (Figure 6A) which is a key characteristic of all eukaryotic Cu-ZnSOD1 [39]. The anti-parallel β-sheets were joined by 3 external loops. The homodimer of cSOD1 exhibits two fold symmetry with the pore size of β-barrel 19 × 12 Å (Figure 6A,B). The active site of each subunits of cSOD1 is oriented in opposite direction relative to other subunit (Figure 6A,B). Each monomer could be coordinated with 2 metals (one Cu^2+^ and one Zn^2+^). The binding pocket of catalytic Cu^2+^ is formed by two loops (Figure 6A).

### 2.5. Similarities Between Structure of Camel SOD1 and Other Mammalian SOD1

**Figure 7:**
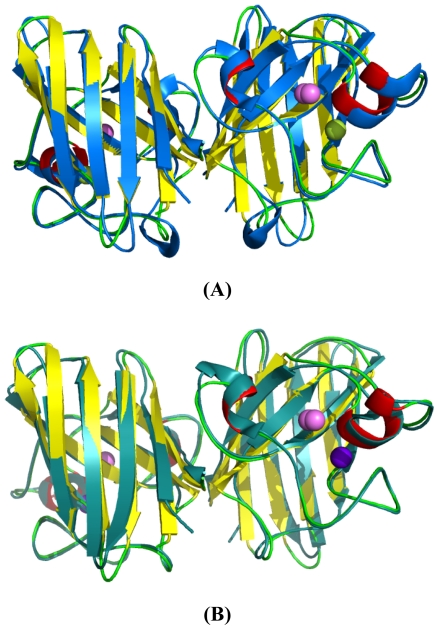
Superimposed 3D structure of cSOD1 *Camelus dromedarius* (yellow) with *Homo sapiens* (blue) (**A**) and *Bos taurus* (deepteal) (**B**). Copper and zinc are shown in purple and green colors, respectively. The superimposition indicated very high similarity between the structures of cSOD1 and the 3D structure of bSOD1 and hSOD1.

**Figure 8:**
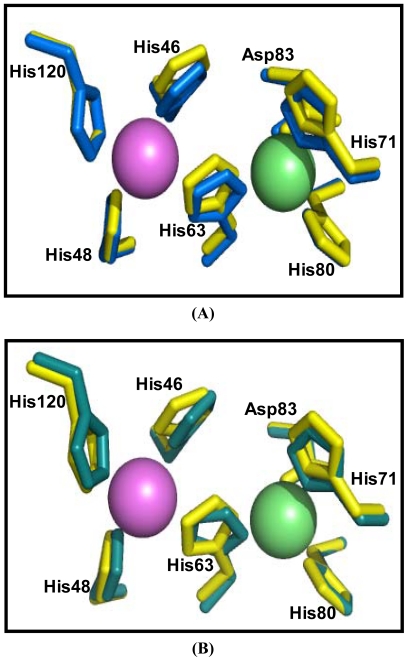
Comparison of the cSOD1 active site residues of *Camelus dromedarius* (yellow) with *Homo sapiens* (blue) (**A**) and *Bos taurus* (deepteal) (**B**). Copper is shown in purple and zinc green color. The active site of the SOD1 from the three organisms contains the highly conserved His and Asp residues. Copper is liganded with four histidines (His 46, 48, 63 and 120) and zinc is liganded with three histidines and one aspartic (His 63, 71, 80 and Asp 83). The residues are numbered according to amino acid sequence of *Camelus dromedarius* (accession number JF758876). This comparison indicated very high similarity between the predicted active site of cSOD1 and that of hSOD1 and bSOD1.

**Figure 9:**
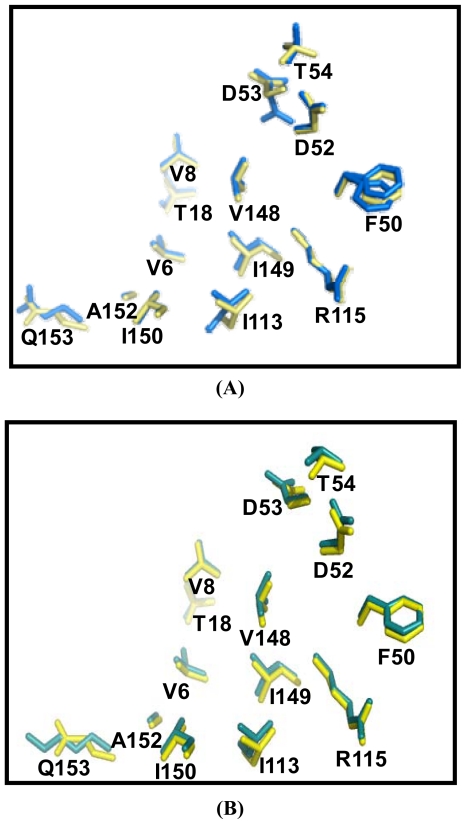
Comparison of the cSOD1 dimer forming residues of *Camelus dromedarius* (yellow) with *Homo sapiens* (blue) (**A**) and *Bos taurus* (deepteal) (**B**). The residues are numbered according to accession number JF758876. This comparison indicated very high similarity between the dimer interface of cSOD1 and that of hSOD1 and bSOD1.

The similarities between cSOD1 and the SOD1 of human and bovine were studied by superimposing their structure in PyMOL program (http://pymol.sourceforge.net) [41]. The overall folds of predicted cSOD1 structure are highly similar to hSOD1 and bSOD1 (Figure 7A,B). The quality of the structural homology was calculated using PDBeFold on EMBL-EBI server [42] (Table 3). When structure of cSOD1 was aligned with the 3D structure of hSOD1, 151 residues were aligned out of 152 input residues. Sequence identities between cSOD1 *vs.* hSOD1 and cSOD1 *vs.* bSOD1 were 87 and 86%. The overall rmsd deviation between cSOD/hSOD1 and cSOD/bSOD1 structure pairs was 0.557 and 0.425, respectively (Table 3). The major structural difference was found in the long flexible loops (Figure 7A,B). The active site as well as dimer contact forming residues in camel, human and bovine SOD1 were superimpose fairly well (Figure 8A,B and Figure 9A,B). The *Q*-score which represents the quality of structure recognition and superimposition indicated that cSOD1 structures had *Q*-score of 0.948 (close to 1.0 means identical structure) when compared with hSOD. Similarly, the superimposed cSOD1 structure with bSOD1 had *Q*-score of 0.961 indicating the very high similarity between the structures of cSOD1 and bSOD1. The *P*-score is used to evaluate the significance of structural similarity. Superimposition of cSOD1 with hSOD1 and bSOD1 had *P-*scores of 24.39 and 19.95, respectively (Table 3). The *Z*-scores of cSOD1 structure superimposition with hSOD1 and bSOD1 were 14.64 and 13.29, respectively (Table 3). Therefore, the values of *Q*-, *P*- and *Z*-scores indicates that the structure of cSOD1 was highly similar to the structures of human and bovine SOD.

**Figure 10:**
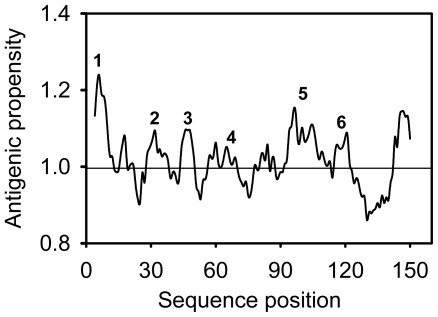
Predicted antigenic properties of cSOD1 using Kolaskar and Tongaonkar method [43]. Six potential antigenic peptides having more than 1.0 antigenic propensity and seven or more amino acids in lengths are predicted.

**Figure 11:**
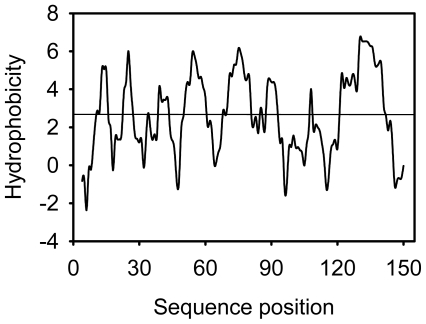
Predicted hydrophobic regions of cSOD1 using the method of Parker, *et al*. [44]. The hydrophobic residues forming the dimer interface are directed under the threshold value and represented by the residues V6, V8, T18, FGDNT (50–54), IGR (113–115) and VIGIAQ (148–153). The electrostatic interacting residues are directed above the threshold line and represented as E132, E133, K136 and T137.

It was predicted also that cSOD1 is highly antigenic as the majority of the protein surface is exposed to the aqueous medium. There are at least six potential antigenic peptides having more than 1.0 antigenic propensity (Figure 10) and seven or more aminoacids in lengths are predicted. The antigenic amino acid sequences and its position are listed (Table 4). The hydrophobic regions of cSOD1 were also predicted (Figure 11). The hydrophobic residues forming the dimer interface are directed under the threshold value and represented by the residues V6, V8, T18, FGDNT (50–54), IGR (113–115) and VIGIAQ (148–153). The electrostatic interacting residues are directed above the threshold line and represented as E132, E133, K136 and T137.

### 2.6. Expression of *cSOD1* Gene by Real Time PCR

**Figure 12:**
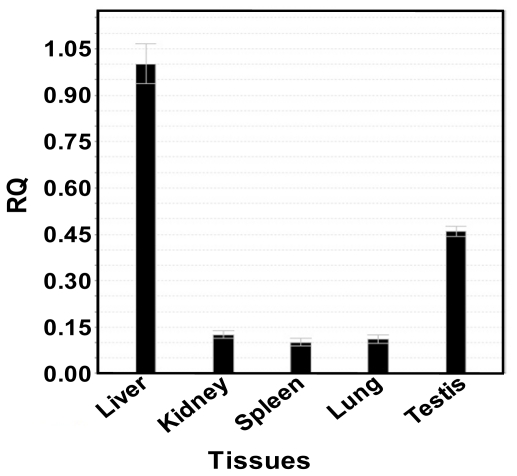
Expression of *SOD1* using Real time PCR and cDNA from different camel tissues. The results are expressed relative to liver as calibrator and using 18S ribosomal subunit as housekeeping gene.

The level of expression of *cSOD1* in different camel tissues (liver, kidney, spleen, lung and testis) was examined by qPCR using a couple of primers that amplify 198 base pairs. The expression of *cSOD1* in liver was taken as calibrator and the expression of 18S ribosomal subunit as endogenous control. The relative expressions of *cSOD1* in kidney, spleen, lung and testis were compared with that of the liver (Figure 12). The highest expression level was found in liver (represented as 100%) followed by testis (45%), kidney (13%), lung (11%) and spleen (10%).

## 3. Discussion

Arabian camel is the most important animal in the Middle East. Despite its economic, and cultural importance, very little biochemical researches are done to elucidate how it can “supersurvive” in the desert’s harsh conditions. Characterization of the cSOD1 in *C. dromedarius* is essential for understanding the impact of exposure to direct sunlight and desert life on the health status of such mammals. There are three different types of eukaryotic SODs namely; the cytosolic CuZnSOD; the mitochondrial MnSOD [13] and extracellular SOD [12]. CuZnSOD (SOD1) is the major type of SOD classes and is responsible for the elimination of superoxide anion produced in the cytosol from the different aerobic metabolic reactions converting it to H_2_O_2_.

The present study is the first work to isolate and characterize the full-length *cSOD1* gene from the one-humped camel. Our results showed amplification of a cDNA fragment of 513 bp covering the coding region of the *cSOD1* using a primer set spanning the gene (Figure 1). This sequence contains the start and the stop codons and part of the 3′ untranslated region. The open reading frame is composed of 459 bp which is comparable with the sequences from most mammalian species (Figure 2). The predicted translation of the open reading frame deduced a protein of 153 amino acid residues of 15.7 kDa and matched several CuZnSOD sequences in GenBank. Our *cSOD1* sequence is submitted in the genbank data base with the accession number JF758876.

Several observations from the primary structure (Figure 2) and from the multiple sequence alignment (Figure 3) merit discussion. First, the primary sequence homology between *cSOD1* and other compared species was greater than 86% (Table 2). This confirms the specificity of the degenerate primer set and places the cSOD1 within the CuZnSOD family. Second, the secondary structure (Figure 5) showed the characteristic 8-β pleated sheets of the CuZnSOD family with a possible very small 9th sheet. The amino acid composition of such sheets favors the formation of the characteristic eight-stranded “Greek key” β-barrel [38] of the known SOD1 class. Third, the presence of the two highly conserved loops, the so-called “electrostatic loop” and the “metal binding” loop in which the enzyme active site is located [45]. The electrostatic loop is located between the residues 122 to 143 which has the characteristic charged amino acids, with the sequence 121-EKPDDLGKGGNEESTKTGNAGSRLA-145. The “metal binding” loop (residues 49–84) contains many of the residues necessary for binding of the metals 49-QFGDNTQGCTSAGPHFNPLSKKHGGPKD(Q)ERHVGDLGNV-87 [46] with the exception of replacing E77 by Q. Fourth, the presence of the characteristic ligands of copper and zinc; copper is bound to four Histidine residues located in His 46, 48, 63 and 120 [47] and zinc is bound by His 63, 71, 80 and Asp 83. Hence, His 63 is shared between the two metals.

Proteins with similar amino acid sequences have a tendency to adopt similar 3D structures. Therefore, it is possible to predict the 3D structure of the putative *C.* dromedarius SOD1, using the recently published *H. sapiens* SOD1 crystal structure as a template for modeling our predicted enzyme [48]. It has been reported that SOD1 is a homodimer composed of two identical subunits tightly joined back-to-back, by hydrophobic and some electrostatic interactions. The 8-stranded “Greek key” beta-barrel sheets of each subunit is arranged into two groups; sheets 1, 2, 3 and 6 have regular length, with little twisting, and are located on the opposite face of the barrel from the active site and sheets 4, 5, 7 and 8 are shorter and more twisted than the other half of the barrel (Figure 6). The β-barrel is a supersecondary structure, found in wide range of enzymes, immunoglobulins and viral capsids [49]. It has been suggested that conserved disulfide bond between residues 57 and 146 greatly increases SOD1 stability [50]. The two cystein residues were found also in *C. dromedarius* at the same position. Disulfide bond formation lowers the conformational entropy. The bigger the length between disulfide bonded cysteines, the larger the entropic contribution to the stabilization of the folded protein structures [51].

The Cu^2+^ is involved in the catalytic activity while Zn^2+^ is in the stability of SOD1 [15]. In the mammalian SOD’s, binding pocket of Cu^2+^ and Zn^2+^ makes distorted tetrahedral geometry (Figure 7A,B). During the catalytic reaction, Cu undergoes cycles of oxidation and reduction which changes its geometry from distorted tetragonal to planar trigonal [45]. The overall quaternary structure, folding, and topology are quite similar to *H. sapiens* SOD1 (Figure 7). The common eight-stranded “Greek key” β-barrel with the electrostatic loop and the metal binding loop were observed.

The two subunits are connected through both hydrophobic and electrostatic interactions facilitated by some amino acid residues. The cSOD1 sequences, showed the same dimer interface sequence as human ones in both the hydrophobic; represented by the residues V6, V8, T18, FGDNT (50–54), IGR (113–115) and VIGIAQ (148–153) and electrostatic interacting residues; represented as E132, E133, K136 and T137 preceded by the conserved catalytically important R143. Superimposition of predicted structure of cSOD1 with two mammalian SOD’s indicated that cSOD1 superimposed with hSOD1 and bSOD1. A *Q*-score of 0.948 and 0.961 was shown when comparing the structure of cSOD1 with hSOD1 and bSOD1, respectively. The value between 0 and 1 indicated the level of similarity. *Q*-score zero means completely dissimilar or unsuperimposed structures, while if the *Q*-score becomes near to 1.0 this represents almost identical structures [52]. The *Q*-score measures the statistical significance of the result relative to an aligned structure. Our results indicated that they were very similar to human and cattle SOD1. The *P*-scores of 24.39 and 19.95, and the *Z*-scores of 14.64 and 13.29, were also mentioned as *Z*-scores of higher than 3.5 means similar structures [53]. This ensures the structure superimposition and high similarity between cSOD1 and both hSOD1 and bSOD1, respectively (Table 3).

SOD1 is ubiquitously expressed, but is present in some tissues at higher concentrations than others [54]. The level of expression of *cSOD1* in different camel tissues (liver, kidney, spleen, lung and testis) is examined using qPCR. The designed primers and the experimental conditions were adjusted to eliminate the primer dimer, self dimer or hairpin form. The expression of *cSOD1* in liver (calibrator) and of 18S ribosomal (endogenous control) was used to measure the relative expressions of *cSOD1* in kidney, spleen, lung and testis (Figure 12). The highest expression level was found in liver followed by testis, kidney, lung and spleen. Our findings suggest that *cSOD1* is highly expressed in liver and testis. The high level in liver is expected where the most of the metabolic processes are performed with the possibility of ROS production. Also it is important to be present in the testis and other tissues of active cell division to avoid any damaging effect caused by superoxide anion.

In conclusion, the degenerate PCR primers designed in this study allowed the amplification of the full-length *cSOD1* from the Arabian camel. CuZnSOD is very similar to human homologue. So, Arabian one humped camel, *C. dromedarius* can be used as a model of studying living in harsh desert conditions as a naturally adapted model. The predicted 3D structure revealed the preservation of several key structural features, such as the metal binding, dimer interface and the 8-beta barrel sheets. The isolated *C. dromedarius SOD1* represents the first full length *cSOD1* gene to be isolated and characterized so far from this unique animal.

## 4. Experimental Section

### 4.1. Samples and Materials

Unless otherwise stated, all *E. coli* strains were grown in LB medium supplemented with 100 μg/mL ampicillin. Liver tissues from three different 2 years old male camel were obtained immediately after killing the animal in Riyadh main slaughterhouse and submerged in RNAlater^®^ solution (Qiagen, Ambion, Courtabeuf, France) to avoid RNA degradation, stored at −20 °C till use.

### 4.2. Oligonucleotide Design

Two degenerated primers were designed from the highly conserved regions of known *SOD1* genes available in the gene bank. These primers are named SODF (forward, 5′-GGATCCATGGCGTTGAAGGCTGTGT-3′) and SODR (reverse, 5′-GGTACCTAGCAAGACAACAGATGAG-3′), respectively. These primers were used in RT-PCR for amplification of SOD-cDNA fragment. On the other hand, two new primer were designed to amplify 198 bp for qPCR namely SODqF 5′-TGGAGACCTGGGCAATGTGAC-3′ and SODqR 5′-CCGCAGGCCAGACGACTTCC-3′, for the forward and reverse respectively.

### 4.3. RNA Extraction, cDNA Synthesis and Reverse Transcription PCR

Fifty mg of liver, kidney, spleen, lung or testis tissue in RNAlater were homogenized in RTL lysis buffer (Qiagen) supplemented with 1% 2-mercaptoethanol, using a rotor-stator homogenizer (Medico Tools, Switzerland). Total RNA was extracted using AllPrep DNA/RNA Mini kit (Qiagen, Cat# 80204), according to the manufacturers’ instruction. Elution was performed with 50 μL nuclease free water. Concentrations and integrity of RNA samples were assessed using NanoDrop-8000 and formaldehyde agarose gel (1%) electrophoresis. Two microgram of the total RNAs were retrotranscribed in single stranded cDNA using ImProm-II Reverse Transcription System (Promega, Cat # A3800,) as recommended by the manufacturer.

### 4.4. Polymerase Chain Reaction and Cloning

Gradient PCR was carried out at annealing temperatures ranged from 50 to 60 °C in a final volume of 50 μL as follow: 25 μL of GoTaq^®^ Green Master Mix (Promega, Cat # M712c), 5 μL of c-DNA, 3 μL of each forward and reverse primers (30 pmole) then the final volume was adjusted to 50 μL with nuclease free water. The PCR condition was 1 cycle at 95 °C for 0:45 min followed by 40 cycles at 94 °C for 30 seconds, 50–60 °C for 45 seconds and 68 °C for 60 seconds. Final extension was carried out at 72 °C for 5 min. The PCR products were analyzed using 1.5% agarose gel by electrophoresis in TAE buffer.

The selected PCR fragment of the expected size was cut from the agarose gel after electrophretic separation and purified using QiAquick gel extraction kit (Qiagen, Cat # 28706), then cloned into the pGEM^®^-T Easy vector (Promega, Cat # A1360). To ligate the generated PCR products onto pGEM-T vector, 2 μL of each purified PCR products were taken in a clean 0.5 mL tube to which 1 μL pGEM-T Easy vector (50 ng) and 5 μL of 2× rapid ligation buffer were added followed by the addition of 3 units of T4 DNA ligase enzyme. The final volume of the ligation reaction was adjusted to 10 μL by the addition of nuclease free water. The ligation mixture was incubated at 15 °C for 16 h. Transformation of *Escherichia coli* JM 109 competent cells was carried out according to Sambrook, *et al*. [55]. The recombinant *E. coli* harboring the recombinant plasmid was screened in selective LB/IPTG/X-gal/Ampicillin/agar plates. Moreover, colonies PCR was conducted to screen recombinant bacteria for ligated DNA insert using T7/SP6 multiple cloning site promoter primers. A small part of each bacterial colony was transferred to a clean sterile Eppendorff tube, to which the rest of the PCR reaction components was added as described earlier. The colony-PCR condition was as follow; 1 cycle at 95 °C for 5 min followed by 30 cycles at 94 °C for 1 min, 50 °C for 1 min and 72 °C for 2 min. The PCR products were analyzed by 1.5% agarose gel electrophoresis.

### 4.5. Studying Gene Expression by qPCR

The expression of *cSOD1* transcripts were studied by qPCR. The reaction was performed three times, each contained cDNA from camel liver, kidney, spleen, lung or testis. The qPCR mixture included the cDNA, 5 pmole each SODqF and SODqR primers and 10 μL Fast-SYBR Green qPCR Master Mix (Applied Biosystems) in a final 20 μL reaction volume as recommended by the manufacturer. The real-time quantitative PCR was performed (Applied Biosystems 7500 Fast real-time PCR system) using the following standard conditions, initial denaturation at 95 °C for 3 min, amplification over 40 cycles of serial heating at 95 °C for 3 seconds and 60 °C for 30 seconds. The amplified product from these amplification parameters was subjected to SYBR Green I melting analysis by increasing the temperature to 95 °C for 15 seconds followed by 60 °C for 1 min and ramping the temperature of the reaction samples from 60 to 95 °C.

### 4.6. Sequencing of the PCR Products and Prediction of Amino Acid Sequence

Sequencing of the PCR product cloned onto pGEM-T Easy vector was carried out according to Sanger, *et al*. [56] using 3130xl Genetic analyzer (Applied Biosystems DNA Sequencing System). The chain termination sequencing reaction was conducted utilizing the ABI BigDyes v3.1 (chain terminator kit as an integral part of the API 3130xl Genetic analyzer) and the T7 or SP6 primers.

The nucleotide sequences were determined in both directions and analyzed using the Seqman PROGRAM [57]. The cSOD1 amino acid sequence was obtained by translating the sequenced DNA fragment using the DNASTAR program [58] and the deduced amino acid sequence was compared with sequences obtained from searches in the NCBI Protein Database using the BLASTP algorithm [59].

### 4.7. Multiple Sequence Alignment and Phylogenetic Analysis

The amino acid sequence of camel SOD1 (accession number JF758876) was used as template to identify similar sequences of other mammalian SOD1 in PSI-BLAST. The homologous sequences from seven different mammals were selected and multiple sequence alignment was performed by ClustalW [34,36]. The output of MAFFT Multiple Sequence Alignment was color coded according to conservancy. The amino acid sequences of cSOD1 and other seven mammalian SOD1 enzymes were used to construct phylogenetic tree using BLOSUM62 [34,36] from MAFFT Multiple Sequence Alignment [34]. The Blast2 seq. was used to calculate the similarities between cSOD1 and other mammalian SOD.

### 4.8. Secondary and 3D Structure Prediction of cSOD1, Superimposition, Antigenicity and Hydrophilicity

The amino acid sequence of cSOD1 was subjected to predict its secondary and 3D structure. The secondary structure was predicted using PSIPRED program [35] while the 3D was predicted using Swiss-model server using homology structure modeling [48]. The structure of cSOD1 was analyzed using PyMOL software (delino Scientific) [41].

The similarities between cSOD1 structure and other mammalian SOD1 structure were analyzed using PyMOL software. The residues involved in active site and dimer formation in cSOD1 and other SOD1 (human and bovine) were superimposed using PyMOL [41]. The quality assessments (rmsd, *P*, *Q* and *Z* scores) of the superimposed 3D structures were done using PDBe on EMBL-EBI server.

The antigenic properties of the cSOD1 were predicted according to the methods of Kolaskar and Tongaonkar method [43]. The antigenicity of peptides was predicted with the more than 1.0 antigenic propensity threshold and more than six amino acid residues. The hydrophobicity of cSOD1 was calculated according to the method of Parker, *et al*. [44].

## Figures and Tables

**Table 1:** Predicted chemical composition of the cloned fragment of cSOD1 using Protean program.

Amino Acid	Number Count	% By Weight	% By Frequency	Amino Acid	Number Count	% By Weight	% By Frequency
A Ala	9	4.06	5.88	M Met	3	2.50	1.96
C Cys	3	1.97	1.96	N Asn	6	4.35	3.92
D Asp	10	7.31	6.54	P Pro	6	3.70	3.92
E Glu	8	6.56	5.23	Q Gln	7	5.70	4.58
F Phe	4	3.74	2.61	R Arg	3	2.98	1.96
G Gly	26	9.42	16.99	S Ser	11	6.08	7.19
H His	9	7.84	5.88	T Thr	7	4.50	4.58
I Ile	9	6.47	5.88	V Val	14	8.82	9.15
K Lys	10	8.14	6.54	W Trp	0	0.00	0.00
L Leu	8	5.75	5.23	Y Tyr	0	0.00	0.00

**Table 2:** Comparison of cSOD1 and other SOD1 enzymes from different mostly similar organisms. The comparison included number of amino acid sequence, percent identity, E-value, isoelectric point (pI) and subunit molecular weight.

SOD	(NCBI Reference Sequence)	No. of Residues	Total Score	Coverage (%)	Identity (%)	Positive (%)	Gap (%)	E-Value	pI	MW
Arabian Camel, *Camelus dromedarius*	AEF32527	153	307	100	100	100	0	6.00E-89	5.94	15.70
Rhesus Monkey, *Macaca mulatta*	NP_001027976.1	154	265	100	87	92	1	2.00E-76	6.22	15.90
Cattle, *Bos taurus*	XP_584414.4	152	263	100	86	93	1	6.00E-76	5.85	15.60
Human, *Homo sapiens*	NP_000445.1	154	261	100	87	90	1	3.00E-75	5.70	15.90
Pig, *Sus scrofa*	NP_001177351.1	153	270	100	88	92	0	7.00E-78	6.32	15.80
Horse, *Equus caballus*	NP_001075295.1	154	246	99	82	89	1	1.00E-70	6.03	16.00
Sumatran orangutan, *Pongo abelii*	NP_001125441.1	155	252	100	85	88	1	2.00E-72	5.87	16.10
Chimpanzee, *Pan troglodytes*	NP_001009025.1	154	261	100	87	90	1	3.00E-75	5.70	15.90

**Table 3:** Pairwise alignment between predicted structure of *Camelus dromedarius* SOD1 with *Homo sapiens* and *Bos taurus* SOD.

Query Structure	Target Structure (PDB)	No. of Residues	Aligned Residues	RMSD	*Q*-Score	*P*-Score	*Z*-Score
Predicted cSOD	hSOD1 (2V0A)	152	151	0.557	0.948	24.39	14.64
Predicted cSOD	bSOD1 (1E9O)	152	150	0.425	0.961	19.95	13.29

**Table 4:** The antigenic amino acid sequences and its position are listed.

No.	Start Position	End Position	Peptide	Peptide Length
1	4	12	KAVCVLKGD	9
2	28	38	PVMVSGSISGL	11
3	44	50	GFHVHQF	7
4	63	69	HFNPLSK	7
5	92	113	GDVAIVSIEDPVISLSGDHSII	22
6	115	122	RTMVVHEK	8
